# *CTLA4CT60* gene polymorphism is not associated with differential susceptibility to pemphigus foliaceus

**DOI:** 10.1590/S1415-47572010005000073

**Published:** 2010-09-01

**Authors:** Márcia Regina Pincerati, Ricardo Dalla-Costa, Maria Luiza Petzl-Erler

**Affiliations:** Laboratório de Genética Molecular Humana, Departamento de Genética, Universidade Federal do Paraná, Curitiba, PRBrazil

**Keywords:** *CTLA4*, *CT60 polymorphism*, pemphigus, fogo selvagem, autoimmunity

## Abstract

Pemphigus foliaceus is an organ-specific autoimmune disease characterized by autoantibodies against the extracellular region of desmoglein 1, a protein that mediates intercellular adhesion in desmosomes. Cytotoxic T-lymphocyte-associated protein 4 (CTLA-4) is a key negative regulator of the T cell immune response, playing an important role in T cell homeostasis and maintenance of peripheral tolerance. Polymorphisms in the *CTLA4* gene have been associated with autoimmune diseases and the functional *CT60* single nucleotide polymorphism (rs3087243, also named *6230G* > *A*) has been proposed to be a casual variant in several of these diseases. The aim of this study was to ascertain whether this polymorphism is associated with inter-individual variation in susceptibility to pemphigus foliaceus. The population sample in this case-control association study comprised 248 patient and 367 controls. We did not found a significant association of pemphigus foliaceus with the *CT60* variants. We conclude that the *CTLA4CT60* polymorphism is not an important factor for pemphigus foliaceus pathogenesis in the population analyzed.

The 2q33 chromosome region harbors genes that encode costimulatory molecules, such as the *cytotoxic T-lymphocyte-associated protein 4* (*CTLA4*), the *cluster of differentiation molecule 28* (*CD28*) and the *inducible co-stimulatory molecule* (*ICOS*), all of which play crucial roles in T cell activation and regulation (Ling *et al.*, 2001).

CTLA-4 is a key molecule expressed by activated T cells that transduces an inhibitory signal after binding to CD80 or CD86 on antigen-presenting cells. CTLA-4 appears to inhibit immune responses by several mechanisms, besides being a critical mediator in peripheral tolerance. Based on its crucial role in immunological homeostasis, *CTLA4* has become one of the main genes of research interest for association studies and has been considered as a target for immunotherapy (Scalapino and Daikh, 2008). Polymorphisms in *CTLA4* are associated with a very wide range of inflammatory and autoimmune diseases (for a review, see Kristiansen *et al.*, 2000; Gough *et al.*, 2005). Several SNPs have been identified in the 2q33 region comprising *CD28*, *CTLA4* and *ICOS* genes (Ueda *et al.*, 2003). The authors found associations between polymorphisms in the segment that includes the 3' region of the *CTLA4* gene with Graves disease, hypothyroidism and autoimmune type I diabetes. Among these polymorphisms, the SNP *CT60G*>*A* (rs3087243, also denominated *6230G*>*A*), was the most-associated marker. The CT60*G allele was correlated with higher disease susceptibility and lower mRNA levels of the soluble alternative splice form of CTLA-4 (sCTLA-4). Recent studies also suggested that polymorphisms in the *CTLA4* gene may influence the development of autoimmune diseases. Palacios *et al.* (2008) concluded that regulatory SNPs in the *CTLA4* gene exert a strong influence on expression levels of both known CTLA-4 isoforms. Moreover, association between *CT60* polymorphism and variation in the frequency of regulatory T cells has been reported. Individuals homozygous for allele CT60*A showed an increase from 30-40% in the frequency of regulatory T cells. Although the basic mechanism connecting the CT60*A allele with an increase in regulatory T cells has not yet been established, these differences reveal a relationship between *CT60* polymorphism and variation in adaptive immune responses (Atabani *et al.*, 2005).

Endemic pemphigus foliaceus (PF), also known as *fogo selvagem* (meaning ‘wild fire'), is an organ-specific autoimmune disease characterized by autoantibodies against desmoglein 1 protein and by loss of adhesion between keratinocytes, leading to intraepithelial blisters of the skin (Warren *et al.*, 2000). Several candidate genes have been analyzed for associations with PF. The *HLA* class II genes (Pavoni *et al.*, 2003) and the *CD40L* gene (Malheiros and Petzl-Erler, 2009) showed the strongest associations. In addition, interactions between some of the genes analyzed have been reported (Martel *et al.*, 2002; Malheiros and Petzl-Erler, 2009). Two SNPs in the *CTLA4* gene, *-318C*>*T* and *49A*>*G*, had been previously analyzed, although no association with PF disease susceptibility was observed (Pavoni *et al.*, 2006).

Based on the reported associations between susceptibility to autoimmune diseases and *CTLA4* polymorphisms, especially the *CT60* SNP, the aim of this study was to extend analysis of this candidate gene to evaluate whether the *CT60* polymorphism is a factor contributing to differential genetic susceptibility to pemphigus foliaceus in the Brazilian population.

We analyzed 248 patients and 367 controls without history of the disease, all unrelated to each other. Diagnosis was according to clinical and histological criteria. Patients and controls were contacted at Hospital Adventista do Pênfigo, Campo Grande, Mato Grosso do Sul State, Brazil. Additional controls were contacted in Curitiba, Paraná State, Brazil. Patients and controls were matched for ancestry. Seventy percent of the individuals were of predominantly European and 30% of predominantly African ancestry. The male:female ratio was close to one, viz. 47% of the patients and 45% of the controls were males. According to age of disease onset, distribution was as follows: from 0 to 9 years, 4.2%; 10-14 years, 8.3%; 15-19, 13.1%; 20-24, 11.3%; 25-29, 9.5%; 30-34, 9.5%; 35-39, 12.9%; 40-44, 8.3%; 45-49, 10.1%; 50-59, 5.4%; 60-69, 4.2%; 70-84, 3.6%. Written informed consent was obtained from all the participants. The study received approval by the Human Research Ethics Committee, in accordance with Brazilian Federal Laws.

Genotyping was performed by polymerase chain reaction amplification followed by restriction fragment length polymorphism analysis (PCR-RFLP). The primers and PCR conditions were the same as those described by Teutsch *et al.* (2004). Allelic, genotypic and allele carrier frequencies (*i.e.*, the frequency of individuals having the allele in either homozygosity or heterozygosity) were estimated by direct counting. Hardy-Weinberg equilibrium was assessed with the Guo and Thompson (1992) method implemented in the ARLEQUIN version 3.11 software package (Excoffier *et al.*, 2005). Comparisons between frequencies in the patient and control population samples were performed by analysis of contingency tables by the chi-square test of independence. The strength of the associations was estimated by the odds ratio (OR), using Woolf's method. The p value of 0.05 was adopted as the significance limit for all statistical tests. The genotype taken as reference was *CTLA4 CT60 G/G*. Therefore, by definition, the OR for this genotype equals 1 and the OR for *A/G* and *A/A* approach the risk of these genotypes relative to the *G/G* genotype.

Allelic, genotypic and allele carrier frequencies are presented in Table 1. Genotype frequencies were in Hardy-Weinberg equilibrium (p = 0.115 and p = 0.829 in patient and control population samples, respectively). No significant association between PF disease status and *CT60* variants was detected.

Associations previously described between susceptibility to some autoimmune diseases and *CT60* polymorphism have been interpreted considering the possible effect of this SNP in the alteration of the ratio of CTLA-4 splicing isoforms, and that elevated levels of sCTLA-4 have already been detected in certain autoimmune disorders (Ueda *et al.*, 2003; Saverino *et al.*, 2007; Kawasaki *et al.*, 2008). In addition, the hypothesis has been raised that sCTLA-4 is involved in control of T cell activation, its levels being regulated by genetic variation in chromosome region 2q33 (Kaartinen *et al.*, 2007). Although several diseases have been associated with *CT60* polymorphism (see above), further studies of the same and other autoimmune diseases in various populations have failed to detect associations, thus concurring with our results (for example, Chang *et al.*, 2007; Tsukahara *et al.*, 2008). One reason for these conflicting results may be differences in allelic and haplotypic frequencies of this marker among populations. Another explanation is that the set of genes contributing to the establishment of different autoimmune diseases is not the same, and that the *CTLA4* gene is important in some diseases but not in others.

In this study, we expanded our previous findings regarding variation in the *CTLA4* gene by analyzing *CT60* polymorphism. Our results lead to the conclusion that genetic variation in *CTLA4* does not play an important role in PF susceptibility and that the effect of variations in this gene differs among autoimmune diseases. We do not exclude the involvement of the CTLA-4 molecule in PF pathogenesis, but show that *CT60* genotypes have no significant impact on pemphigus foliaceus disease susceptibility.

## Figures and Tables

**Table 1:** Genotypic, allele carrier, and allelic frequencies for the *CT60* SNP in patients and controls.

	Frequency (%)				
	Patients (n = 248)	Controls (n = 367)	OR^1^	95% CI^2^	p^3^
Genotypes					
*G/G*	37.9	33.2	1		
*A/G*	43.1	49.6	0.76	0.53-1.09	0.14
*A/A*	19.0	17.2	1.03	0.65-1.64	0.89
Allele carriers					
*G+*	81.0	82.8	0.89	0.58-1.35	0.57
*A+*	62.1	66.8	0.82	0.58-1.14	0.23
Alleles					
*G*	59.5	58.0	1.06	0.84-1.34	0.62
*A*	40.5	42.0	0.94	0.75-1.19	0.62

^1^Odds ratio; ^2^Confidence interval; ^3^Probability values.
